# Effects of Familiarity and Dialect Experience on the Description of Tonal Variant

**DOI:** 10.3389/fpsyg.2022.787048

**Published:** 2022-06-21

**Authors:** Huangmei Liu, Dafydd Gibbon

**Affiliations:** ^1^College of Foreign Languages, University of Shanghai for Science and Technology, Shanghai, China; ^2^Faculty of Linguistics and Literary Studies, Bielefeld University, Bielefeld, Germany

**Keywords:** familiarity, dialect experience, cross-dialect perception, subjective description, tone perception

## Abstract

This study investigates the tonal variant description of the official dialect in China (Putonghua) as a factor in the coevolution of dialects. Three sociophonetic factors, target tone familiarity, tonal variant familiarity, and tonal inventory size, are included in order to raise interesting theoretical questions concerning the role of familiarity and dialect experience in sound change. Standard Putonghua tones are manipulated in height and shape in order to create systematically varying stimuli. Speakers from three Chinese dialect groups, Beijing Mandarin, Shanghai Wu, and Guangzhou Cantonese, are invited to rate the applicability of a description of pitch contour and height to the stimuli. The three dialects have different tonal inventory size, and their native speakers have different levels of familiarity with Putonghua tone or Putonghua tonal variants. The above three sociophonetic factors make different predictions about listeners' performances. The findings of the experimental analysis of data confirm the role of tonal variant familiarity in predicting participants' descriptive decisions on tone height variants. Tonal variant familiarity is also combined with tone inventory size to explain the assignment of descriptions of tone shape variations. This suggests that when variations still follow the phonetic pattern of the tone distribution of the Putonghua tonal system, listeners give phonetic patterns the primary role in acoustic decisions but still benefit from their dialect experiences in making more precise acoustic decisions. It also suggests that when variations violate the phonetic features of the target tonal system, they may depend on familiarity with the individual variant. This study applies an innovative sociophonetic method by conducting a perception experiment online with a self-paced procedure. The findings here are crucial for examining the relationship between sociophonetic factors and listeners' acoustic decisions and the cultural coevolution of cross-dialect tonal variation. The findings here also give support to the validity of the current web-based crowd perception experiment design and are also needed to facilitate research under restricted conditions, such as a pandemic situation.

## Introduction

Cultural coevolution of languages and dialects refers to cultural influences such as contact on language change, as opposed to language internal pressures on language change, such as assimilatory effects on sounds in context and their consequent push or pull effects on other contrasting sounds. The cross-dialect description refers to the descriptive decision of listeners when hearing and perceiving other non-native dialects. It is strongly associated with perception behavior and subject to listeners' knowledge of the target dialect as well as their experience in their own dialect. For each national language, there is at least an official variety or dialect and other regional dialects. The official dialect and regional dialects sometimes coexist and evolve independently, but often they develop more interactively. Strictly speaking, an official dialect has no native speakers but is spoken by a large number of speakers of various regional dialects. Thus, as a consequence of the coevolution of official dialects and local dialects, there is a certain number of regional variants of an official dialect (Liu et al., 2020). Therefore, how regional natives perceive and describe the variants of an official dialect is a source of the coevolution of the official and regional dialects in a local region. Research on the description of variations in an official dialect contributes to broadening theories of dialectal tone coevolution across different sociolinguistic factors.

The Chinese official dialect (Putonghua) is the officially designated version of Mandarin with the aim of standardizing regional accents and increasing intelligibility in cross-dialect communication. Like dialects in other countries with many regional dialects, such as France, Germany, and the United States, Putonghua also has a huge amount of variations across Chinese regions. Putonghua tones vary dramatically and systematically (or at least show some regional patterns) across regions (Chen et al., 2003; Jin et al., 2008), which make the language an ideal target for studying the influence on the perception of tonal variants by sociolinguistic factors. Here, in particular, we focused on the role of familiarity with the official dialect and dialect-specific tone system in a perception-based tonal variant description task. This study applies an innovative sociophonetic method involving an online perception experiment with a self-paced procedure. It allows this research to access a much greater number of participants, and to provide listeners with an authentic crowd perception environment (as opposed to laboratory experiments) at the same time. The findings here are crucial for establishing the relevance of sociophonetic factors and listeners' acoustic decisions for the coevolution of cross-dialect tonal variation. The design may also give support to the validity of web-based crowd perception experiment design in facilitating research under restricted conditions, such as a pandemic situation.

### Chinese Official Dialect and Regional Dialects

In the Chinese dialectological tradition, regional language groups are called dialects. It should be realized, though, that differences between some of these dialects are large enough to make the dialects mutually unintelligible (Tang and van Heuven, 2009). Since the 1990s, Putonghua has been popularized and has spread throughout China and started to have an impact on regional dialects. At the same time, Putonghua evolved into local variants shared by regional groups, leading to a three-tier pattern: Putonghua, Putonghua variants, and regional dialects. Since then, Putonghua and regional dialects have been coevolving into an intimate “dance” of speech variants in a cultural crossover context.

Acoustic research on changes has been very fruitful and has demonstrated a significant impact of Putonghua on dialects (Tang and van Heuven, 2009, 2015; Wu et al., 2014; Liu et al., 2020). A large number of studies on dialectally accented Putonghua tonal variants have been made (Chen et al., 2003; Jin et al., 2008). Putonghua and the dialects are consequently in constant coevolution. Therefore, this study takes advantage of the complexity of the situation of Chinese dialects in order to examine how listener-related sociophonetic factors affect sound change perception.

The three Chinese dialects concerned are Beijing Mandarin (BM), Shanghai Wu (SW), and Guangzhou Cantonese (GC), which are representative of the three major east coast dialect families: Northern Mandarin (Beijing Mandarin), Wu (Shanghai Wu), and Cantonese (Guangzhou Cantonese), respectively. The map in the Appendix shows the location of the three dialects. Table 1 provides an overview of the tones of BM (Shi, 2002), SW (Xu and Tang, 1988), and GC (Chen, 2011). Tone transcriptions are based on Chao's (1930) 5-digit description system.

**Table 1:** Tone systems of Beijing Mandarin, Shanghai Wu, and Guangzhou Cantonese (with tones' traditional names in italic).

**Dialects**	**Tones**								
Putonghua/Beijing Mandarin	55 *Yinping*	35 *Yangping*	214 *Shang*	51 *Qu*					
Shanghai Wu	53 *Yinping*	34 *Yinqu*	13 *Yangqu*	5 *Yinru*	12 *Yangru*				
Cantonese	55/53 *Yinping*	21/11 *Yangping*	35 *Yinshang*	13 *Yangshang*	33 *Yinqu*	22 *Yangqu*	5 *Shang Yinru*	3 *Xia* *Yinru*	2 *Yangru*

Putonghua tones vary both in pitch height and contour shape in daily communication, partly because of internal language factors, such as coarticulation and tone sandhi (Rose, 1990) and partly because of external factors, such as the influence of gender, social status, and contact with other dialects (Kurpaska, 2010). Some variants are documented by impressionistic studies based on the transcription of monosyllabic words in citation form (Lin and Wang, 1985; Wang, 1990; Chen et al., 2003; Li et al., 2003; Liu, 2004; Yu et al., 2004; Jin et al., 2008; Jin, 2010; Cao, 2012; Ye, 2012) and are listed as follows:

1. *Tone Yinping* has variants such as /55/, /44/, /33/, and even /22/. Its pitch realizations are reported to be more likely to vary in height.
2. *Tine Yangping* has variants such as /35/, /34/, /25/, and even /24/. Its pitch realizations could vary in contour slope and mean pitch height.
3. *Tone Shang* has variants such as /214/, /212/, /324/, and even /434/, and it seems to mainly vary in pitch height.
4. *Tone Qu* has variants such as /51/, /41/, /31/, and even /21/, and its pitch realization could vary in contour slope and mean pitch height.

Therefore, it is interesting to explore how these variants are perceived and described by listeners of different dialect backgrounds. This question is closely connected with the mutual influence between the official and regional dialects since the variants influence local speakers' cognitive processes of perception and further behavior of production. This study was, thus, motivated by the question of finding differences in listeners' descriptions of tonal variants when they are from different dialectal groups that contribute to the understanding of dialectal tone coevolution across different sociolinguistic factors. Therefore, in this study, stimuli are designed to vary in pitch height and contour shape in order to simulate several types of variants in search of answers to the question above.

According to the characteristics of the regional dialect and local listeners, the three dialectal groups show three major differences as follows:

1. Different sizes of tonal inventory: BM has four tones, and its tonal system is almost the same as that of Putonghua. SW has five tones, while GC has nine (refer to Table 1 for citation tones of the three dialects).
2. Local populations have different degrees of familiarity with Putonghua and Putonghua tones: BM speakers use Putonghua in most daily communication and in almost all media and schools. SW speakers also have a very high frequency of hearing and using Putonghua, since Shanghai has a large migrant population. The majority of media and schools also use Putonghua, although SW is still used to a certain extent because Shanghai has a local dialect based on TV and radio, Shanghai schools have a large number of local teachers, and some schools even have a course teaching SW. GC speakers are the least familiar with Putonghua among the three because GC is used as the major communication language in Guangzhou and is very popular on local TV and radio and in schools. The sociocultural environment has consequently reduced the chances of exposure to Putonghua tonal variants for SW and GC speakers, especially for the latter, in contrast to BM speakers.
3. Local populations have different degrees of familiarity with Putonghua tonal variants: when there is category-to-category assimilation between variants of the standard Putonghua tone and the regional tone, we suggest that the local populations are familiar with the variant that would facilitate local speakers' description of tonal variants. Therefore, besides inventory sizes, the existence of category-to-category assimilation between dialectal tones and Putonghua tonal variants would also affect tone perception and tone description. Such assimilations are affected by the distribution of tones in different height register areas and the shape of the local tones. GC has nine tones (refer to Table 1), with a large number of flat and contour tones over all three registers (high register, mid register, and low register), which consequently provide a wide choice of tones for the assimilation of Putonghua tonal variants (refer to **Figure 2**). BM has a tonal system that is exactly the same as standard Putonghua tones and provides itself with four straightforward category-to-category assimilations. SW has five tones (and 2 checked tones which should be excluded), which make it comparably less assimilable to the Putonghua tonal variants mentioned above.

### Familiarity and Cross-Dialect Perception

Familiarity with the target dialect contributes to a cross-dialect perception of sound change and consequent convergence (Sumner and Samuel, 2009; Pickering and Garrod, 2013; Walker and Campbell-Kibler, 2015; Pardo et al., 2017). For example, Sumner and Samuel (2009) proved that experience played a crucial role in a cross-dialectal lexical recognition task. Listeners from another dialect process the out-of-dialect variants of standard words, and those who have previous exposure were better. The Automatic Motor theory (Goldinger, 1998; Pickering and Garrod, 2013) proposes automatic imitation of what is heard in order to make perception decisions. Therefore, the more listeners are familiar with a language variety, the more accurately they are likely to perceive it. Ross et al. (2021) compared two listeners groups' perceptual sensitivity with phonetic convergence in their own dialect and another dialect. The two listener groups from the same two regions with that in Sumner and Samuel (2009) have different levels of familiarity with the other dialect. The acoustic materials are vowels. The result did not support the suggestion that greater experience with a dialect affects the perception of phonetic convergence. The contradictory results from the above two experiments might be due to the difference in processing task, since the former was about lexical recognition, while the latter was about phonetic similarity. Thus, it suggests that the relationship between familiarity and cross-dialect perception is not yet clear.

This study inquires about how listeners from the three dialectal groups perform in their perception of tonal variants of the official dialect. The task in this study is an acoustic decision about the target stimuli, which is close to the recognition tasks in Sumner and Samuel (2009). Thus, the Automatic Motor theory predicts that familiarity contributes significantly to the tonal variant description here. Therefore, the Automatic Motor theory suggests that better familiarity with Putonghua in daily usage (refer to Chinese Official Dialect and Regional Dialects) makes the BM group perform best in the tonal variant description, followed by the SW group and finally the GC group.

However, in this study, there is another kind of familiarity, tone variant familiarity. The expression “tone variant familiarity” in this study specifically refers to the existence of a similar tone category in the native dialect to the tonal variants created as stimuli. This kind of familiarity has its roots in dialect experience. It is through dialect experience that the local speakers acquire acquaintance with and sensitivity to the stimuli that are similar to their tonal categories. There is a considerable quantity of supportive evidence on the effect of category-to-category assimilation on prosody perception, with the function of explaining the influence of L1 tones on learning the tones of another tonal language (Best, 1995; Best et al., 2001). Therefore, different degrees of familiarity with Putonghua variants (refer to Chinese Official Dialect and Regional Dialects) provide another order in terms of success in describing tonal variants for the three groups, with GC as the best, then BM, and finally SW as a general idea. Consequently, the roles of two kinds of familiarity in cross-dialect perception need further investigation.

### Dialect Experience and Cross-Dialect Prosody Perception

Theoretical frameworks for cross-language suprasegment perception include the L2 Intonation Learning theory (Mennen, 2015), Native Language Magnet model (Kuhl, 2004), Communication Accomodation theory (Giles, 1973), and Perceptual Assimilation Model for Suprasegmentals (PAM-S) (So and Best, 2008, 2010). Although the prestige role of Putonghua results in a form of bilingualism among Chinese speakers, L2 approaches are only indirectly relevant to the Putonghua-dialect relationship. This study is concerned with prosody, in particular lexical tones. These theories suggest a role of L1 prosodic experience in L2 prosody perception and emphasize the influence of L1 on categorical perception in L2 prosody perception.

The prestige role of Putonghua makes the majority of Chinese bilingual-like: most people speak the official language and a dialect that could be substantially or slightly different from the official language. A considerable number of studies on cross-dialect perception unveiled the influential role of dialect experience on tone perception (Gandour, 1984; Xu et al., 2006; Peng et al., 2010). Peng et al. (2010) found that in a rising tone and falling tone perception task, Cantonese dialect speakers had almost the same category boundary width for speech and nonspeech continua, while Putonghua-only speakers had two significantly different ones, and both of them were significantly sharper than that of German participants. Peng et al. (2010) argued that it is the richer tone inventory of Cantonese that strengthened its speakers' ability in pitch perception. In other words, research of this kind suggest that a larger tonal inventory size makes its speakers more sensitive to pitch perception.

The three dialects of concern in this study have noticeable different tonal systems with different tonal inventory sizes (refer to Chinese Official Dialect and Regional Dialects). According to the above assumption concerning tonal inventory size, the GC group will perform best in Putonghua tonal variant description tasks, then it is the SW group that is the first and the BM group is the last. Such a prediction contradicts with the previous two kinds of predictions based on the familiarity that is mentioned in Familiarity and Cross-Dialect Perception. Therefore, the experimental design gives an opportunity to test which factor predicts better tone perception performance in this study: *target tone familiarity, tonal variant familiarity*, or dialect *tonal inventory size*. Detailed research questions and hypotheses are explained in the next section.

## Methodology

### Stimuli

**Figure 1:**
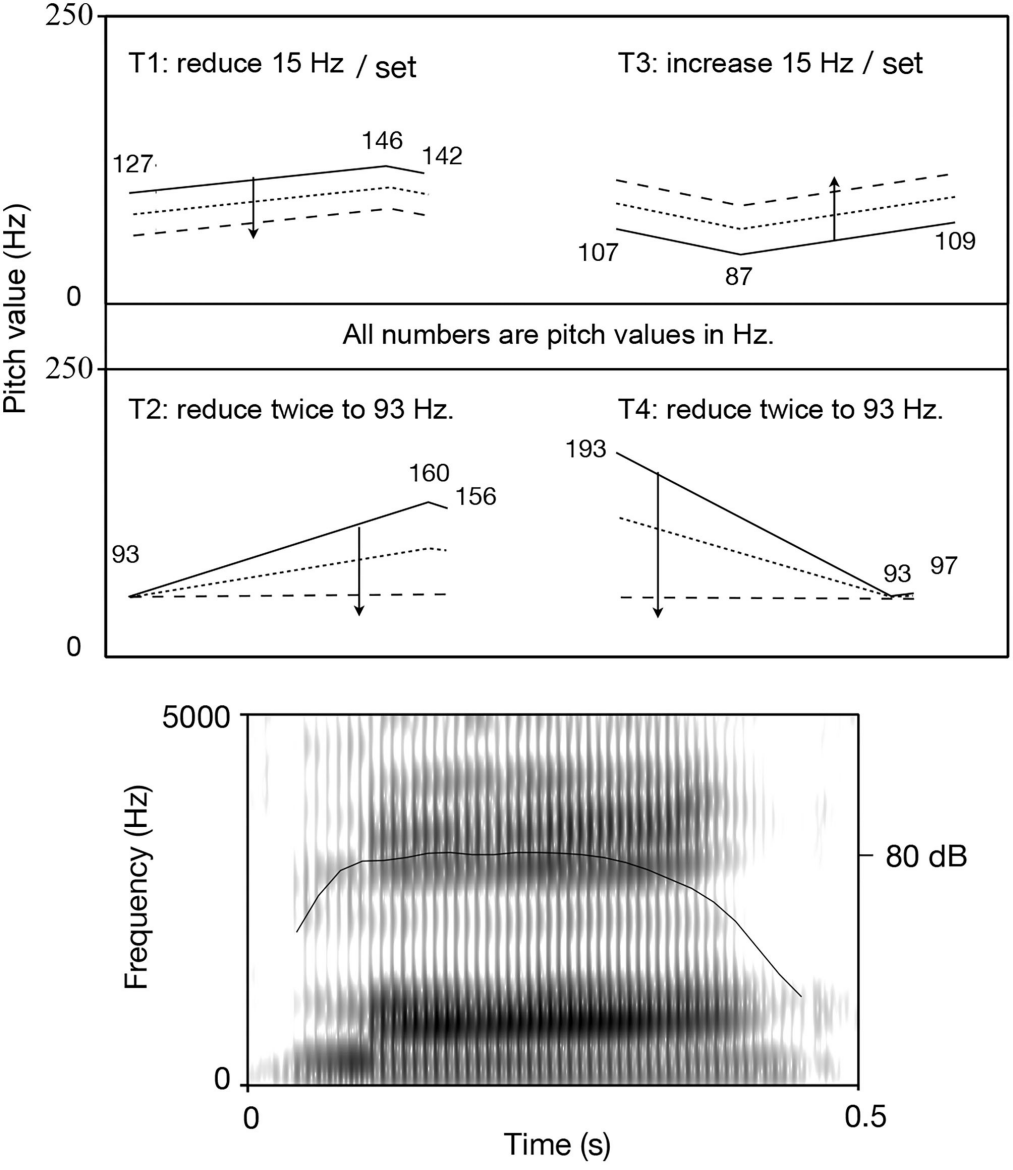
Generation of stimuli: manipulated tonal contours (set 1: solid line, set 2: dashed line, set 3: dotted line; the arrow shows the manipulating direction; pitch value is marked out for the original curve) (upper), and the spectrogram of the Putonghua syllable /ma/ for generation of stimuli (lower).

A 36-year-old male university lecturer who has an A-level Putonghua certification from the Chinese National Putonghua Test was recruited to produce original sound files for this study. The male speaker was asked to produce each Putonghua monosyllabic lexical word with 10 repetitions and pause in citation context, ordered from T1 to T4 in a soundproof booth with a Sennheiser professional headset (chin-fixated microphone). The recording was conducted by Praat with a MacOS laptop with sample rate at 44.1 kHz and 16-bit resolution. Fundamental frequency (F0) was estimated by the autocorrelation method provided in Praat (Boersma, 2001), setting appropriate F0 cut-offs. Pitch values of three pivot points for each tone were extracted to represent the tone (Liu, 2020). For T1 (a level tone) and T2 (a rising tone), the sequence of extreme value points is “Low- High-Low.” For T3 (a dipping tone) and T4 (a falling tone), the sequence is “High-Low-High” (refer to Figure 1). Tone duration was defined as the time interval between the first and the third points, the extreme time values. Such quantification methods for tones were proved to be capable of maintaining tonal information well (Liu et al., 2020, in press). The quantification method is based on Chao's (1930) impressionistic description method for Mandarin tones and proposes that three points are enough to describe a tone. The average pitch values in Hz were calculated at the three points.

The stimuli are designed to vary in pitch height and contour shape to simulate the two types of variants (refer to Chinese Official Dialect and Regional Dialects). The three sets of stimuli of T1 simulate documented variants of tones /55/, /33/, and /22/, and the three variants of T3 simulate documented variants of tones /214/, /324/, and /434/. T1 and T3 mainly vary in pitch height. The three sets of T2 simulate variants of tones /35/, /34/, and /33/^1^. The three sets of T4 simulate its variants of /51/, /31/, and /11/^1^. Figure 1 illustrates the generation procedure of two types of tone variant simulation, and Table 2 describes the three sets of stimuli in terms of *high, mid*, and *low*.

1. Manipulation of height: the whole original contour of T1 was lowered twice, and the whole contour of T3 was increased twice, resulting in two sets of two stimuli. Each manipulation was by 15 Hz, a small but still perceptually distinct difference. Every time, the manipulation of height was done by a change of 15 Hz, which is a small but still perceptually distinct difference (Peng et al., 2010). However, we were quite cautious about whether the difference among set 1, set 2, and set 3 could trigger a categorical difference in perception, since this would need experimental evidence, and binary decisions on categorical perception are not immediately relevant to the research question on sociophonetic scalar assimilation trends. Therefore, we designated set 1 as the original set of tones, while set 2 simulates the minor variants and set 3 simulates the major variants.
2. Manipulation of shape: the offset of T2 and the onset of T4 were lowered in the direction of low-level tones (refer to Figure 1). After the first manipulation, T2 and T4 still maintained the original contour (rising and falling). The second manipulation changed the tone shape completely and made them low-level tones, which are different tonal categories compared with the original tones.^2^
3. All the speech stimuli in the present study were resynthesized by superimposing the manipulated tonal contour on the same Putonghua syllable /ma/ (500 ms) with high level tone with the pitch-synchronous overlap and add (PSOLA) method (Moulines and Laroche, 1995) in Praat (Boersma and Weenink, 2022).

**Table 2:** Description of stimuli.

**Set 1**				**Set 2**				**Set 3**			
**T1**	**T2**	**T3**	**T4**	**T1**	**T2**	**T3**	**T4**	**T1**	**T2**	**T3**	**T4**
High level	High rising	Low falling-rising	High falling	Mid level	Mid rising	Mid falling-rising	Mid falling	Low level	Low level	High falling-rising	Low level

**Figure 2:**
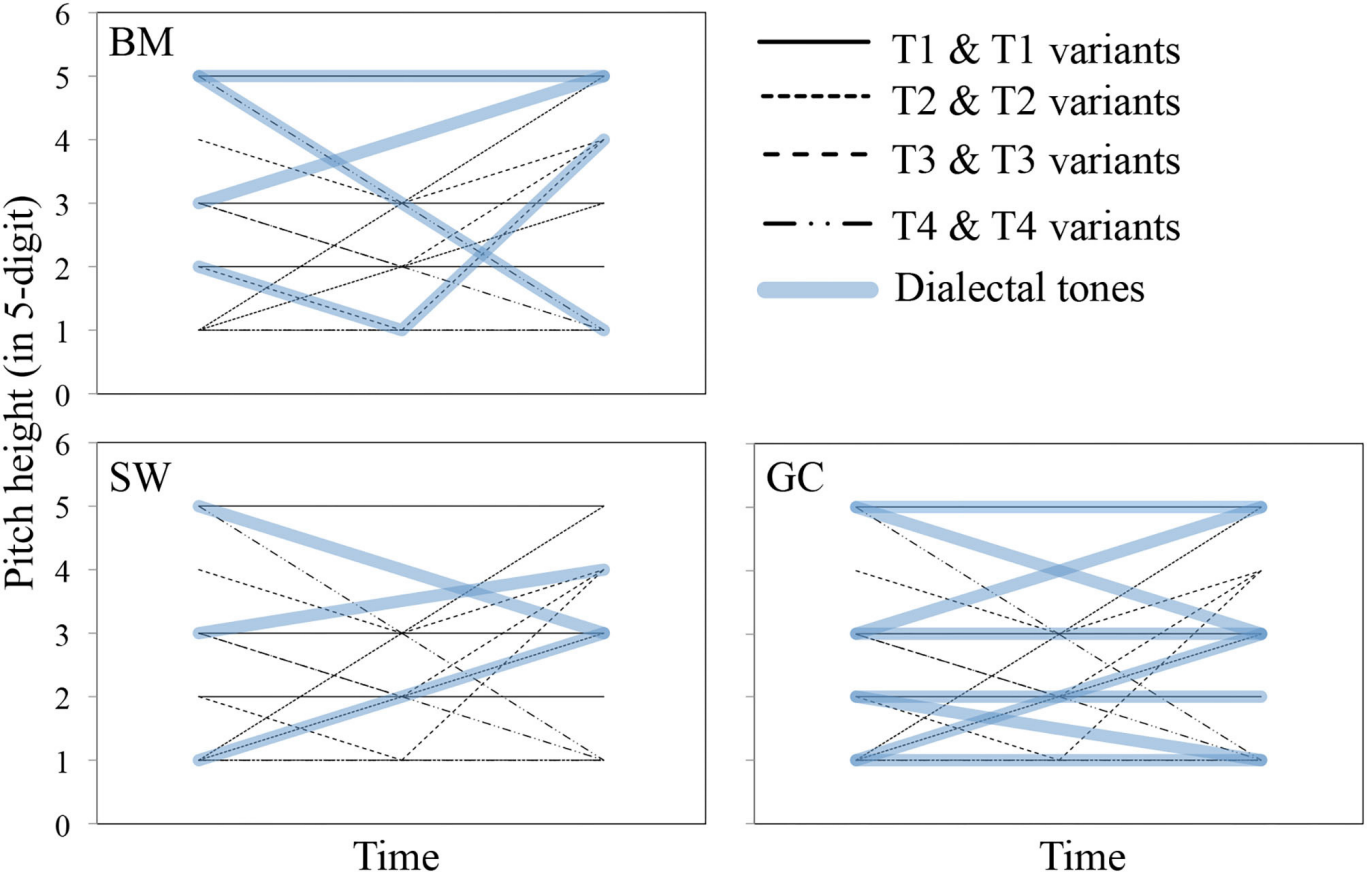
Different similarity levels of the three stimuli sets and dialectal tones of the three regions (Beijing Mandarin, BM, upper left; Shanghai Wu, SW, lower left; Guangzhou Cantonese, GC, lower right) suggesting different levels of familiarity with the stimuli by the three regional populations.

The pitch of the stimuli is represented by a 5 digit scale. The stimuli are visualized together with the regional Putonghua tones shown in Figure 2. The upper left figure compares the stimuli and BM tones and shows 3 pairs of overlapped tone curves (T1 set 1 and BM *yinpin*, T3 set 1 and BM *Shang*, T4 set 1 and BM *Qu*) and 1 pair of similar curves (T2 set 1 and BM *yangpin*). The lower left figure shows the stimuli and SW tones, with 1 pair of overlapped tone curves (T2 set 2 and SW *yangqu*) and 1 pair of similar curves (T2 set 1 and BM *yinqu*). For the stimuli and GC tones, the lower right figure shows 5 pairs of overlapped tone curves and 2 pairs of similar curves, with no counterparts for the stimuli of T2 set 2, all T3 sets, and T4 set 2. It provides evidence that local tones have various similarities to the stimuli in this study that permit comparisons with local speakers with different levels of familiarity with the stimuli.

### Research Questions and Hypotheses

We tested the participants' description of height and shape of the three sets of stimuli. We proposed a set of research questions: whether the description accuracy of (a) tone height variation and of (b) tone shape variation could be predicted by (c) tone familiarity, (d) tonal variant familiarity, or (e) tonal inventory size using the present sociophonetic methods.

As mentioned above, altogether, there are three predictions to be tested. Predictions from the two models about the results of the present task are contradictory. Predictions 1 and 2 are both based on “familiarity facilitates sensitivity” assumptions. The third prediction is based on “larger tonal inventory size increases tone perception sensitivity” assumptions.

Prediction 1: tone familiarity causes perceptual accuracy on both tone height and shape changes to increase from the GC group to the SW group and to the BM group.

Prediction 2: the similarities of tone categories between the three stimuli sets presented in Table 3 show that seven GC tonal categories are similar to Putonghua stimuli, more than the four between BM and Putonghua. The comparisons shown in Figure 2 also support this prediction. SW only has two tonal categories that are similar to Putonghua. Therefore, tonal variant familiarity is predicted to cause a decrease in perceptual accuracy on both tone height and shape changes from the GC group to the BM group and to SW group. However, it also predicts poor performance for the GC group with the T3 stimuli.

**Table 3:** Tone categories from the three dialects similar to the present tone stimuli.

**Stimuli**	**Similar tone categories from dialects**		
	**BM**	**SW**	**GC**
T1_set1_	High level		High level
T1_set2_			Mid Level
T1_set3_			Low level
T2_set1_	High rising	High rising	High rising
T2_set2_			
T2_set3_			Low level
T3_set1_	Low falling-rising		
T3_set2_			
T3_set3_			
T4_set1_	High falling	High falling	High falling
T4_set2_			
T4_set3_			Low level

Prediction 3: The GC group performs best, profiting from its largest tonal inventory size among the three, followed by the SW group and finally the BM group.

### Participants

Altogether, 177 participants from the three dialect groups were recruited: 42 BM locals, 60 SW locals, and 75 GC locals. Some speakers (9 BM, 4 GC, and 9 SW participants) were excluded because of incomplete results. In total, 157 participants (*n*_*BM*_ = 33; *n*_*SW*_= 51; *n*_*GC*_ = 73) were analyzed in this study. The BM participants were aged 20 to 55 (mean = 36.31; SD = 10.05; *n*_*female*_: *n*_*male*_ = 16: 17). The GC participants were aged 20 to 53 (mean = 24; SD = 8.6; *n*_*female*_: *n*_*male*_ = 44: 31). The SW participants were aged 21 to 52 (mean = 33.34; SD = 6.26; *n*_*female*_: *n*_*male*_ = 27: 33). Gender ratio was controlled to <1:1.5.

The metadata about the priority of Putonghua in daily life were collected before the task. The participants were required to choose yes or no about whether Putonghua plays the most important role in their daily life. All the BM participants chose “yes”; 28 out of the 51 SW participants (35%) and 27 out of the 73 GC participants (27%) chose “yes.” The metadata result is consistent with the claim in the literature mentioned in Chinese Official Dialect and Regional Dialects that Putonghua exposure decreases from BM to SW to GC. Putonghua priority shows no relationship with participants' age or gender. Participants with any hearing deficits are asked to not do the test at the beginning of the test page. To avoid any unforeseen problems by personal sound loudness preference, the participants are allowed to adjust the loudness volume of the sound files on their equipment.

### The Task and Procedure

We designed a novel sociophonetic survey of the ascription of pitch description to tones, in which listeners rate the applicability of descriptions of pitch contour and height to recordings. The rating task is to listen to one monosyllabic token at a time and to respond in each case with meta-phonetic assignments of pitch descriptions to these tokens in a 5-point Likert format. A typical assignment claim is *The tone of the sound is level*, and the selections are 5: *yes*, 4: *maybe*, 3: *not sure*, 2: *maybe not*, and 1: *no*. Listeners can listen to each token as many times as they want.

For the pitch *contour* (*shape*) description, the experiment provides *level, rising, falling-rising, falling*, and *rising-falling*. The descriptor *rising-falling*, which does not apply to Putonghua, was designed as a distractor. For the pitch *height* description, the experiment provides *high, mid*, and *low*. The study uses the online survey software *OSCAR-Online Survey Collation and Reporting* (Gibbon and Liu, 2018), which has been successfully used in the sociophonetic teaching context and provides convenient remote access to sociophonetic surveys during the present epidemic period and permits distributed survey administration. The method had been used successfully in previous studies. Gibbon and Liu (2018) investigated whether listeners' dialect affiliation can be predicted from their subjective meta-phonetic description of Putonghua tones in naturally uttered stimuli with the monosyllabic carrier [ma] using the same online survey design. This design succeeded in clustering listeners from the same dialect region into the same group with an accuracy of above 85%. This justifies the use of the *OSCAR* tool for this study.

There are 12 sound files, each of which is followed by eight statements for the participants to rate. There are five ones for *shape* (*level, rising, falling-rising, falling*, and *rising-falling*) and three for *height* (*high, mid*, and *low*). The 12 sound files are tested in three blocks, with each set presented as a block. The task asks the participants to listen to one file at least twice. Then, it asks the participants to rate each description statement at a time. Statements 1–8 share the same template such as:

The tone of the sound is *level*. Rating task buttons: 5, 4, 3, 2, or 1.

## Results

Descriptive statistics are examined first about the distribution of the data. The accuracy data were submitted to the Linear Mixed Effect Model (LMM) under Type III tests with *region* as the fixed factor and *age* as the random effect factor. Fixed-effects predictors smaller than 0.05 are considered as significant. The experimental results are compared with the three kinds of predictions. The out-of-prediction results are recognized as odd ones and are given special attention. Altogether, 15,072 responses were collect from the 157 participants; 5,652 were for *height*, and 9,420 were for *shape*.

### Height Description Results

#### Descriptive Statistics

The descriptive statistics of the participants' accuracy in the description of tone height are shown in **Table 5**. Similar standard deviation for each stimulus by each region was found, which indicates that a slight difference in age distribution did not produce unbalanced data under the present sociophonetic method. The descriptive statistics suggest that the GC group achieved a good mean accuracy in most types of stimuli (except for T4_set2_). The BM group kept achieving a high accuracy rate in description of the original stimuli (except for T1_set1_). However, the SW group seemed to perform the worst for all types of the stimuli, except for T4_set1_. With exceptions, the above results are highly consistent with prediction 2 based on *tonal variant familiarity* (refer to Research Question and Hypotheses).

#### Difference Analysis

**Figure 3:**
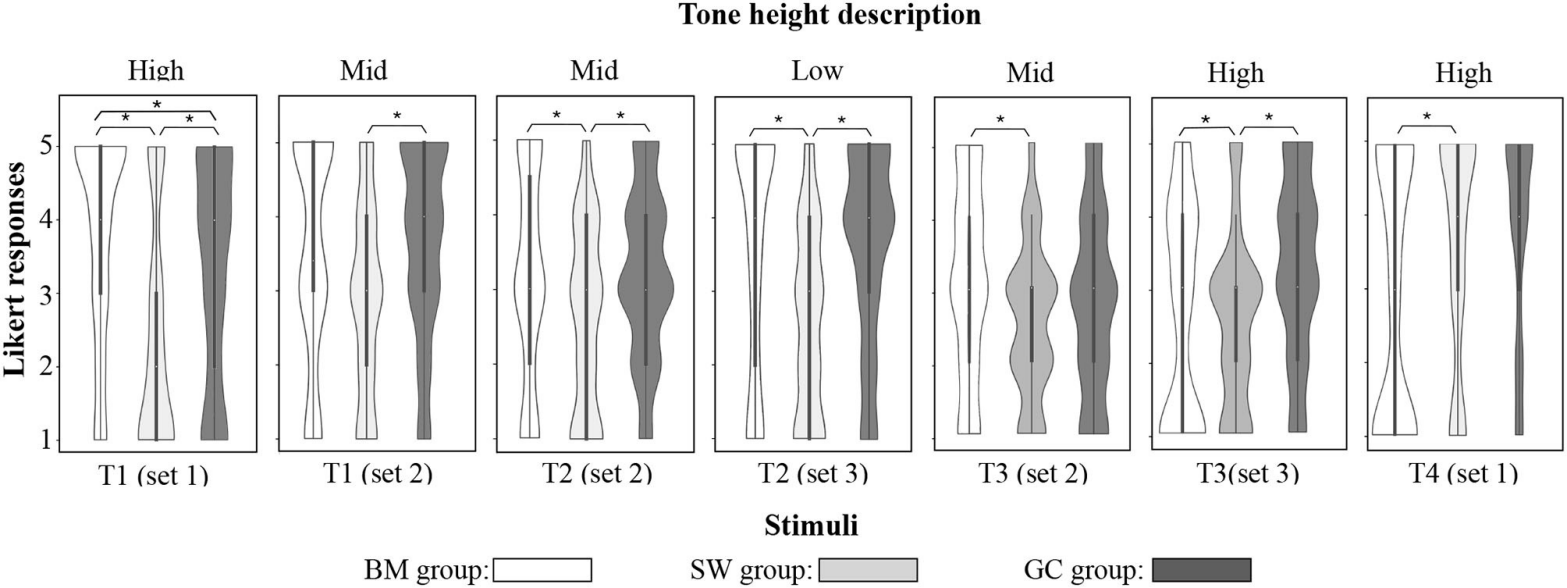
Distribution of tone height description for stimuli with significant between-group differences. Responses were separated by dialect group and tone set.

The present LMM analysis identified a significant between-dialect-group difference in description of T1_set_ 1 (*p* < 0.001, *F* (2, 153) = 11.53), T1_set2_ (*p* = 0.02, *F* (2, 153) = 3.92), T2_set2_ (*p* = 0.01, *F* (2, 153) = 5, T2_set3_ (*p* < 0.001, *F* (2, 153) = 9.13), T3_set2_ (*p* = 0.049, *F* (2, 153) = 3), T3_set3_ (*p* = 0.029, *F* (2, 153) = 3.64), and T4_set1_ (*p* = 0.045, *F* (2, 153) = 3.16), with a significantly better accuracy rate from the GC group and the BM group. *Post hoc* Tukey pairwise comparisons with Holm-Bonferroni adjustments showed that the GC group performed significantly better than the SW group in T1_set1_ (*p* = 0.001), T1_set2_ (*p* = 0.006), T2_set2_ (*p* = 0.004), T2_set3_ (*p* < 0.0001), T3_set1_ (*p* = 0.033), T3_set2_ (*p* = 0.048), and T3_set3_ (*p* = 0.027). It also showed that the BM group performed significantly better than the SW group (*p* < 0.0001) and the GC group (*p* = 0.05) in T1_set1_. Figure 3 shows the distribution of tone height description responses for stimuli with significant between-group differences. The superior capacity of accurate description could be seen through the density of responses in the zone of [3, 5]. No significant interactions between age and participant groups were found during the statistical analysis.

#### Odd Results for the Predictions

Still, two strangely unsatisfying results (T1_set3_ and T4_set3_) for the GC group, predicted to be successful in Table 3 by the category-to-category assimilation, remain to be explained. Reasons may be found in the undergoing tone merger process for Cantonese that the two variants for *yinping* (21 and 11) are merging and are quite close to *yangqu* (22) (refer to Table 1).

### Shape Description Results

#### Descriptive Statistics

**Table 5** shows the descriptive statistics of the participants' choice of tone shape variants. Generally the description accuracy for all the shape descriptions is rather successful, except the one for T1_set3_, for all the three regional groups. Also, similar sizes of derivation have been noticed here. Still, odd results are discovered and are listed below.

#### Difference Analysis

**Figure 4:**
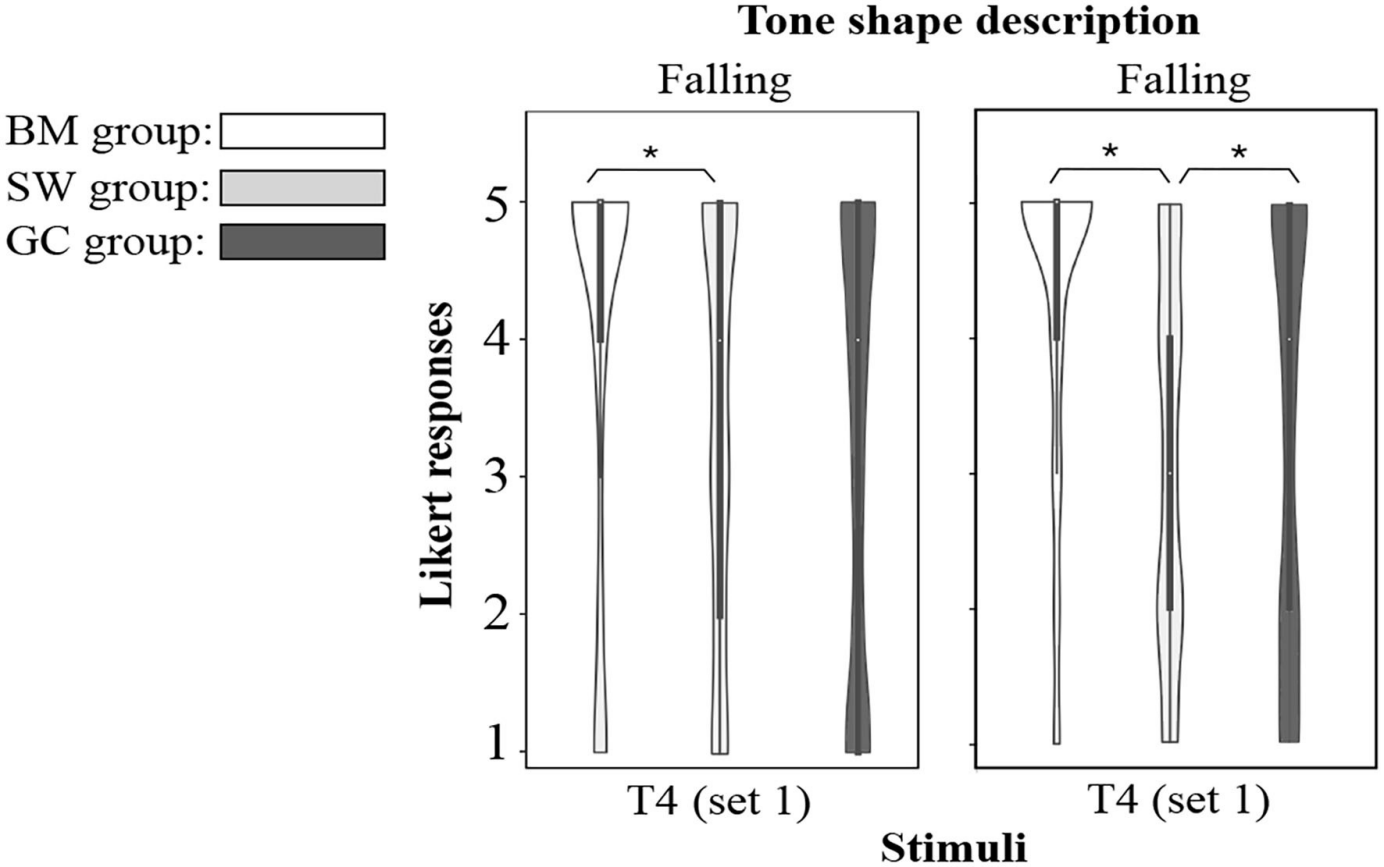
Distribution of tone shape description responses for stimuli with significant between-group difference. Responses were separated by dialect group and tone set.

The linear mixed effect model only identified two significant between-dialect-group different stimuli: T4_set1_ (*p* = 0.004, *F*(2, 153) = 3.73) and T4_set2_ (*p* = 0.004, *F*(2, 153) = 5.77), with a smaller size of standard derivation for BM group data (refer to **Table 5**). *Post hoc* Tukey pairwise comparisons with Holm-Bonferroni adjustments showed that the BM group performed significantly better in T4_set1_ than the SW group (*p* = 0.035) and the GC group (*p* = 0.015), and in T4_set2_ better than the GC group (*p* = 0.014) and the SW group (*p* = 0.001). T4_set1_ is a high falling tone, and T4_set2_ is a mid falling shaped tone. Table 1 and Figure 1 together give some hints to explain these discoveries, and that both of them are more close to BM T4 in the acoustic realization. Although it did not cause them being misperceived, it indeed facilitated the BM participants to be more precise and sure with their decisions. Figure 4 shows the distribution of tone shape description responses for stimuli with significant between-group differences. The BM participants' superior capacity of accurate description could also be seen through the density of their responses at “5.” No significant interactions between age and participant groups were found during the statistic analysis.

#### Odd Results for the Predictions

Two types of odd results did not conform to the expectations of the prediction. The first type was T1_set3_, which was a *level* tone (see Table 2). The descriptor “*level*” was not favored by participants from the BM and GZ groups. Therefore, we checked all the shape descriptor's results and found that most participants from the two groups prefered “*rising*” for T1_set3_. The means of “*rising*” by the BM group and the GC group (mean_BM_ = 3.48, sd_BM_ = 1.54; mean_SW_ = 2.8, sd_SW_ = 1.36; mean_GC_ = 2.92, and sd_GC_ = 1.51) were obviously higher than those of “*level*.” Paired T-tests between “*rising*” and “*level*” were conducted within each regional group, which showed that the difference between “*rising*” and “*level*” of the BM group was significant, while that of the SW group was also significant but for “*level*” higher than “*rising*” (*p*_BM_ = 0.009; *p*_SW_ < 0.001; *p*_GC_ = 0.06). This result might be due to moving the whole curve to lower frequency zone making the gentle slope of T1_set3_ more audible (refer to Figure 1 for slope information).

The second type of odd result was about all the absent predictions in Table 3. Empty cells predict bad perception. However, participants from all three groups played very well in all of them. This might suggest that the participants could perceive the tone shape variants very well using their knowledge of Putonghua. Also, the fake distractor “rising-falling” was extremely expelled by the participants, which also supported this assumption.

### Difference Analysis for Stimuli Sets

#### Description Results for Stimuli Sets

**Figure 5:**
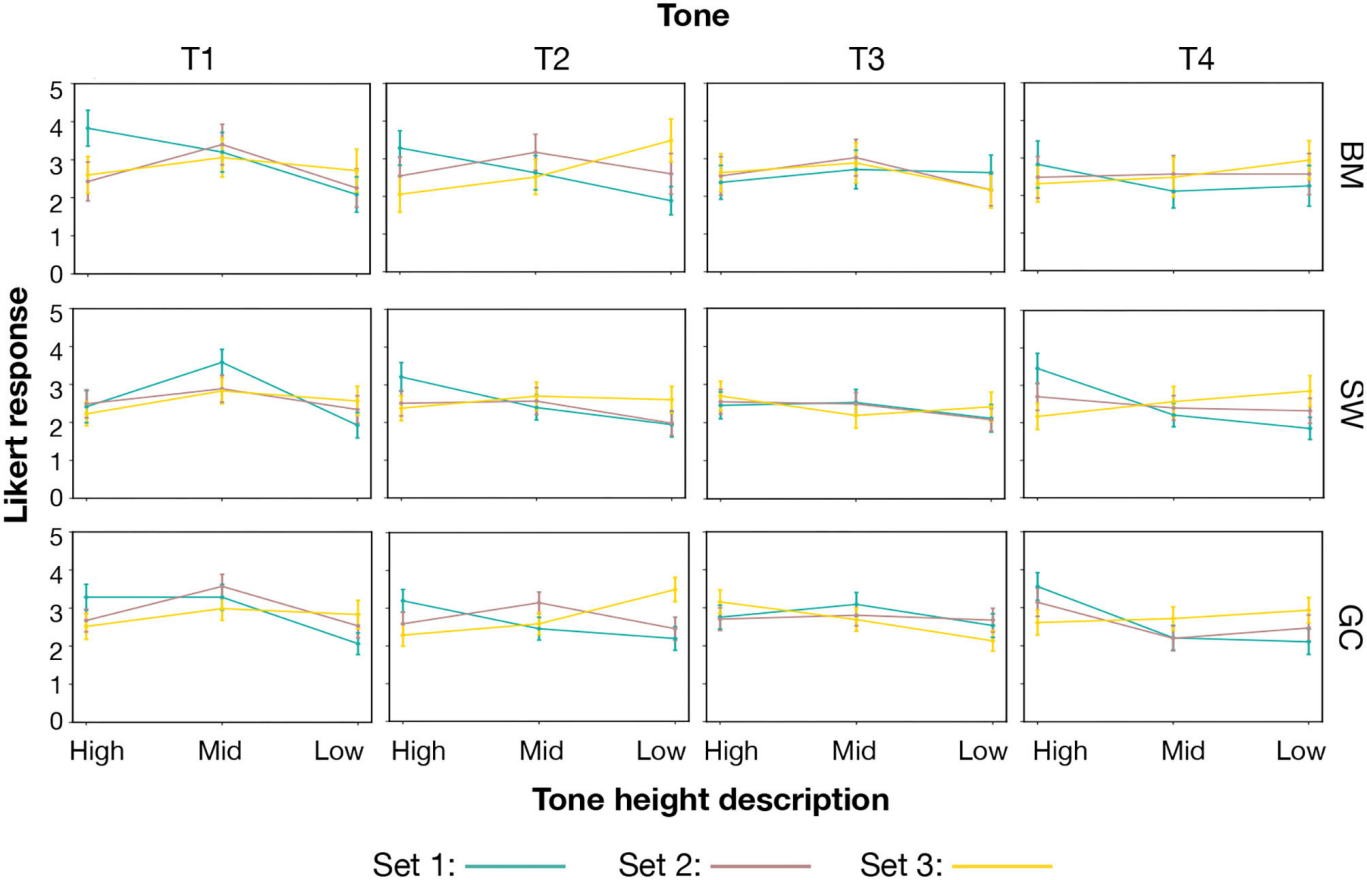
Set curve for height descriptors separated by tone and dialect group.

**Figure 6:**
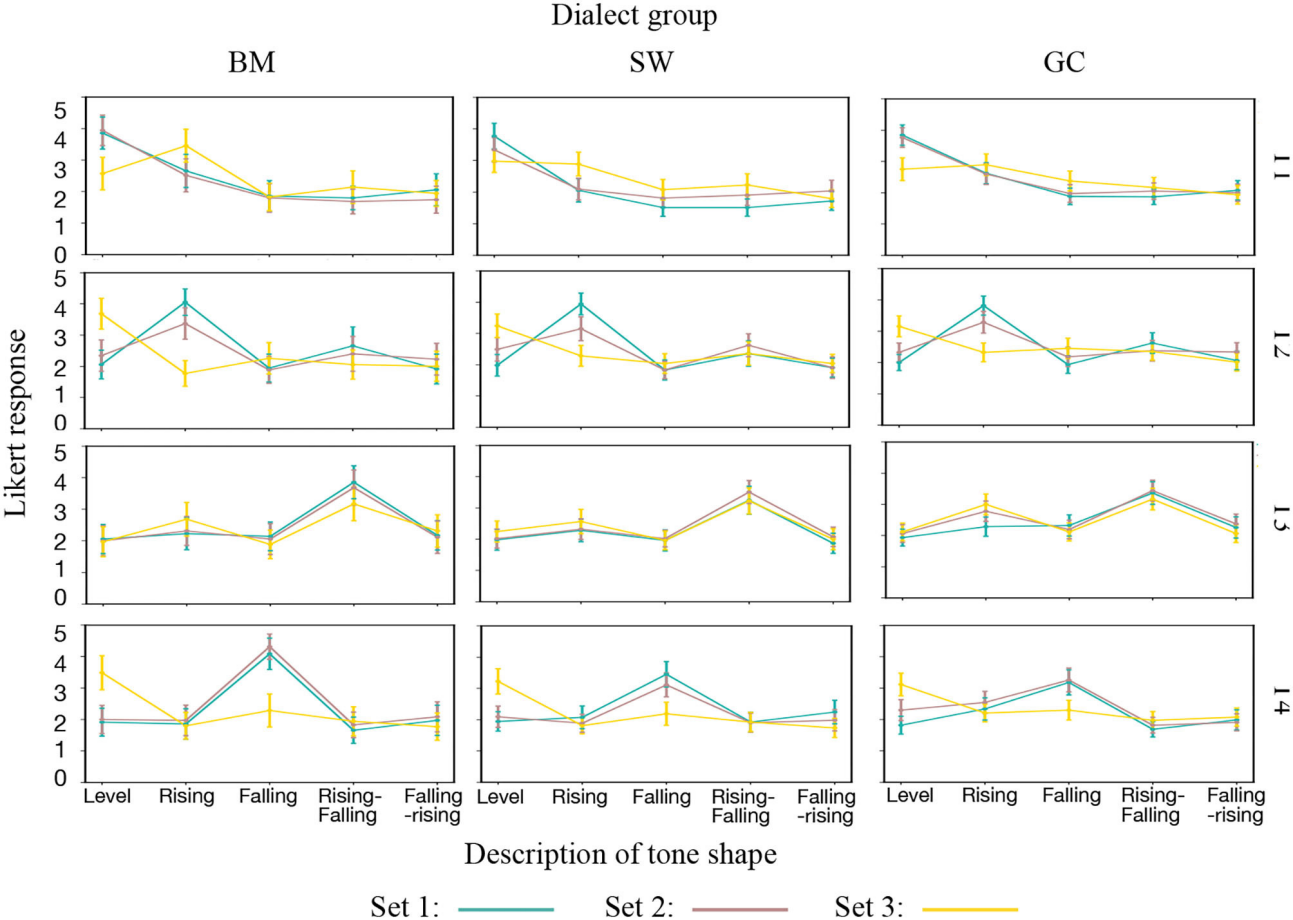
Set curve for shape descriptors separated by tone and dialect group.

The description results for the height and shape descriptors of each stimulus are shown in Figures 5, 6.

#### Analysis of Variance

A two-way repeated measures analysis of variance (ANOVA) with the mixed effect model was carried to determine the impact of dialect group and tone stimuli set on height description decision. Dialect group is the between-subject factor, with tone stimuli set as the within-subject factor. All effects were reported as significant at *p* <0.05.

For T1, T2 and T3, significant main effects of dialect group (T1: *F* (2, 154) = 8.32, *p* < 0.001, *p*η^2^ = 0.10; T2: *F*(2, 154) = 7, *p* = 0.001, *p*η^2^ =0.08; T3: *F*(2, 154) = 6.07, *p* = 0.003, *p*η^2^=0.07) were found. However, no significant effect of dialect group was found on T4 results [*F*(2, 154) =0.48, *p* < 0.62, *p*η^2^ = 0.006]. Strong significant main effects of set were found in T1, T3, and T4, and a marginally significant main effect of set were found in T2 [T1: *F*(2, 308) = 7.38, *p* = 0.001, *p*η^2^ = 0.05; T2: *F*(2, 308) = 2.36, *p* =0.1, *p*η^2^ =0.015; T3: *F*(2, 308) = 4.53, *p* = 0.012, *p*η^2^ = 0.029; T4: *F*(2, 308) = 14.19, *p* < 0.001, pη2 = 0.084]. The BM group is always good at set 1 for all the four tones.

There was a strong interaction effect between dialect group and stimuli set for T1 and T2 but quite marginally for T3 and T4 [T1: *F*(4, 308) = 2.76, *p* = 0.028, *p*η^2^ = 0.035; T2: *F*(2, 154) = 2.58, *p* = 0.038, *p*η^2^ = 0.032; T3: *F*(1, 154) = 1.37, *p* = 0.24, *p*η^2^ = 0.018; T4: *F*(1, 154) = 1.94, *p* = 0.1, *p*η^2^ = 0.025]. Along with the interaction pattern shown in Figure 5, it indicates that the SW group played rather uneasily for all three sets, with noticeable overlaps between sets 1 and 2 and comparatively flat curves for each set. It only achieves good scores for set 1 of T2 and T4. It indicates the low sensitivity to the stimuli of the SW group. Meanwhile, the interactive patterns still support that the GC group and the BM group achieved significantly better accuracy than the SW group for most of the stimuli (the differences can be found in Tables 4, 5).

**Table 4:** Means for tone height description accuracy with standard deviation in parentheses.

**Stimuli**	**BM group**	**SW group**	**GC group**
T1_set1_	3.88 (1.36)	2.39 (1.55)	3.26 (1.51)
T1_set2_	3.42 (1.58)	2.88 (1.28)	3.62 (1.33)
T1_set3_	2.7 (1.69)	2.55 (1.40)	2.9 (1.61)
T2_set1_	3.3 (1.33)	3.22 (1.39)	3.25 (1.30)
T2_set2_	3.18 (1.40)	2.55 (1.29)	3.18 (1.19)
T2_set3_	3.52 (1.70)	2.59 (1.27)	3.6 (1.31)
T3_set1_	2.61 (1.37)	2.08 (1.29)	2.6 (1.34)
T3_set2_	3.03 (1.42)	2.43 (1.08)	2.88 (1.14)
T3_set3_	2.61 (1.48)	2.69 (1.41)	3.25 (1.31)
T4_set1_	2.82 (1.88)	3.43 (1.51)	3.66 (1.55)
T4_set2_	2.55 (1.44)	2.37 (1.17)	2.23 (1.41)
T4_set3_	2.94 (1.52)	2.84 (1.50)	2.96 (1.46)

**Table 5:** Means for tone shape description accuracy with standard deviation in parentheses.

**Stimuli**	**BM group**	**SW group**	**GC group**
T1_set1_	3.79 (1.56)	3.73 (1.52)	3.92 (1.34)
T1_set2_	3.88 (1.47)	3.27 (1.43)	3.85 (1.28)
T1_set3_	2.55 (1.52)	2.98 (1.29)	2.77 (1.56)
T2_set1_	4 (1.3)	3.84 (1.31)	3.9 (1.3)
T2_set2_	3.27 (1.51)	3.33 (1.51)	3.08 (1.4)
T2_set3_	3.73 (1.44)	3.19 (1.4)	3.2 (1.4)
T3_set1_	3.79 (1.6)	3.42 (1.55)	3.18 (1.66)
T3_set2_	3.61 (1.69)	3.49 (1.44)	3.45 (1.36)
T3_set3_	3.18 (1.59)	3.14 (1.5)	3.24 (1.53)
T4_set1_	4.15 (1.44)	3.22 (1.73)	3.4 (1.51)
T4_set2_	4.27 (1.23)	3.23 (1.66)	3.06 (1.42)
T4_set3_	3.52 (1.6)	3.22 (1.56)	3.16 (1.49)

For shape analysis, the same two-way mixed design repeated measures analysis of variance (ANOVA) was conducted. No significant main effects were found for dialect group or set, or for interaction between dialect group and set. The set curves in Figure 6 for all the three groups on all the tones have similar patterns, which also supported the findings in 3.2 that participants from any of the three groups can perceive the shape variants well.

Therefore, generally, the results here favor prediction 2 based on the *tonal variant familiarity* that the GC group outperforms SW, and is marginally better than BM. The BM and SW groups tend to be better at set 1 than at sets 2 and 3, while the GC group tend to be better at both sets 1 and 3 than at set 2.

## Discussion

In this study, a perception-based tone description task was conducted to investigate the relationship between Putonghua (the official dialect in China) tonal variant description and three listener sociophonetic factors: *tone familiarity, tonal variant familiarity* and *tonal inventory size*. *Tonal inventory size* was adopted in order to develop predictions for the influence of native dialect experience on results. Daily exposure and usage was adopted to qualify *tone familiarity*. Category-to-category assimilation between the native dialect tone and the stimuli was introduced as *tonal variant familiarity*. Three dialectal groups of participants took part in the experiment.

Potential differences for descriptions on tonal variants are brought in by the sociophonetic differences of the three groups, BM, SW, and GC, because the three dialects of concern have a different *tonal inventory size*, and their native speakers have different levels of familiarity to the Putonghua tone or to the Putonghua tonal variant referring to the category-to-category assimilation between native dialect tone and the stimuli. The difference in the above three sociophonetic factors is predicted differently in listeners' performance. *Tonal inventory size* predicts an increase in advantages of dialect experience from the BM group to the SW group to the GC group both with height and shape variant perception. *Tone familiarity* predicts better performance from BM than SW, and the worst from GC. However, *tonal variant familiarity* predicts an accuracy decreasing order from the GC group to the BM group to the SW group. Therefore, it provides an interesting and suitable context for testing which sociophonetic factor was more plausible to account for the decisions on description of cross-dialect tonal variants. It contributes to the whole picture of the cross-dialect coevolution of tones based on tone variant perception from a sociophonetic perspective.

### Familiarity vs. Native Dialect Experience

The first research question asked which sociophonetic cue could predict tone height variant description more precisely. Better predictions by *tonal variant familiarity* were borne out by the higher accuracy of the GC group when compared with the other two groups and the lowest accuracy of the SW group. The GC group has seven category-to-category assimilations between the stimuli and the Guangzhou Cantonese tones. All this contributed to the success of the GC group. Meanwhile, the SW dialect group did not perform better than the BM group, which did not meet the expectation based on *tonal inventory size*. However, *tone familiarity* seems to have contributed rather little in describing the tone height variants used in this study.

The second research question asked which sociophonetic cue could prediction tone shape variant description more precisely. The results deny any of the predictions in the following aspects: (1) significant differences in results in all three sets of the four tones were not found among the three groups; (2) all the participants yielded positive results and showed capability of perceiving and making the right acoustical description decision on all the present designed shape variants. A plausible reason for this was that all the participants were well-taught on the phonetic patterns of the Putonghua tones by attending Chinese courses in school. Therefore, when doing the task, they used their knowledge of the Putonghua phonetic patterns of the tone distribution to make descriptive decisions. In this case, they all succeeded in the task. Another evidence supporting this proposed reason was the extremely low score for the distractor “rising-falling.” Higher consistency of acoustic description decisions were also evidenced by the BM and GC participants' less dispersed description response than that of the SW participants.

We also investigated the three sociophonetic cues' role in perceiving the three stimuli sets. Generally, the results here favor the prediction based on *tonal variant familiarity*, since the GC group outperforms the SW group and is only marginally better than the BM group. The BM and SW groups tend to be better at set 1 than at sets 2 and 3, while the GC group tends to be better at both sets 1 and 3 than at set 2. This finding further explored the role of sociophonetic factors in cross-dialect tone variant perception. Peng et al. (2010) found that in a rising tone and falling tone perception task, Cantonese dialect speakers had almost the same category boundary width for speech and nonspeech continua and argued that it is the richer tone inventory of Cantonese that strengthened its speakers' ability in pitch perception. Furthermore, we here found how bigger *tonal inventory size* facilitates its speakers to be more sensitive in tone variant description by supporting its speakers with more category-to-category assimilation between the stimuli and its native tones. It is why the Shanghai speakers with larger *tonal inventory size* did not outperform the Beijing Mandarin speakers here.

The expression “*tone variant familiarity*” in this study narrowly refers to the existence of a similar tone category in the native dialect to the tone variants created as stimuli. Actually, it is found to be a robust cue when participants perceive and make description decisions on cross-dialect tonal variants. Although it is considered to be a phenomenon of familiarity, in fact, it has its roots in dialect experience. There is a considerable quantity of supportive evidence on the effect of category-to-category assimilation on prosody perception, with the function of explaining the influence of L1 tones on learning the tones of another tonal language (Best, 1995; Best et al., 2001). We took a further step to prove that the present design of description of cross-dialect tonal height variant is relevant to the existence of the category-to-category assimilation between the target tone and the native tone. The results here also indicate that greater experience with Putonghua might not affect prosodic descriptive decisions on tonal variants. Ross et al. (2021) also found a similar failure of familiarity in predicting cross-dialect segmental phonetic convergence perception. Both our research and Ross et al. (2021) argued that this might be due to a certain level of familiarity having already been achieved through social environments such as media and education (Sumner and Samuel, 2009; Walker and Campbell-Kibler, 2015), whereas under conditions of a completely unfamiliar language, exposure still had great influences (Green, 1998; Abutalebi and Green, 2008; Grainger et al., 2010; Declerck et al., 2017).

### Tonal Variant of the Official Dialect

Putonghua is the official dialect that has the largest speaker population. A dialectally accented Putonghua tone has been found to have various phonetic differences, while tonal contrasts were maintained well across dialectal regions (Chen et al., 2003; Jin et al., 2008). The character of accented Putonghua has similarities with heritage Cantonese. Heritage Cantonese has been recorded as only different at phonetic level compared to Hong Kong Cantonese speakers (Chang et al., 2011; Tse, 2016). Kan et al. (2019) also found the relevance of category-to-category assimilation to the development of tone perception of heritage Cantonese speakers who were born and raised in the United States.

Two kinds of variant were created by the present experiment for Putonghua tones, height variant and shape variant. According to the above discussion, in the manipulation of *shape*, tonal contrasts were maintained. Therefore, it was easy for the all the participants to use their knowledge of Putonghua to solve the questions well. Although the slopes of T2 and T4 were manipulated, no new types of tones were created. However, *height* variations violate the tonal contrast. In Putonghua, there is no contrast as *high, mid*, or *low*, because although a large amount of variations in pitch values of each tones was noticed at the individual level, speakers would not connect it with phonemic contrast of *height*. In other words, listeners might have noticed a T1 uttered with a low pitch frequency or a T3 uttered with a high pitch frequency. However, they usually did not recognize it as a contrast. Thus, requiring listeners to make decisions on manipulated height variants was beyond their stored knowledge of Putonghua tone, which forced them to be cautious and highly dependent on category similarity with their native tones (refer to Height Description Results).

### Influence From Native Dialect Evolution

An interesting finding in this investigation was that the ongoing tone-merge in the native dialect of GC speakers showed an impact on height variant description. T1_set3_, T4_set2_, and T4_set3_ were predicted to be successful for the GC listeners (see Table 3) by the category-to-category assimilation. T1_set3_ and T4_set3_ were low level tones (like 11/22 in Chao's five-digit description), which assimilate a variant of GC *yinping* (11) and quite close to GC *yangqu* (22) (refer to Table 1). T4_set2_ was a mid falling tone (like 31 in Chao's five-digit description), which assimilated the other variant of GC *yinping* (21). However, there is an undergoing tone merger process for Cantonese that the two variants for GC *yinping* (21 and 11) are merging and are quite close to GC *yangqu* (22) (Chen, 2011; Ou, 2012). This could make the GC listeners uncertain when making a decision, because they will find it harder to find the native category to assimilate. Tone merging was also reported to reduce discrimination accuracy on acoustically similar tones in Cantonese (Ching, 1984; Ciocca and Lui, 2003; Kan et al., 2019). Therefore, influence of native dialect tone merging on cross-dialect tonal variant description is proposed here. More studies will be needed to know the precise mechanisms involved.

### Description Task: Perception or Production?

In this experiment, we designed a metaphonetic tone description task as a method for collecting data of language variation perception. The high degree of relevance between the present results and the selected sociophonetic factors indicates that the present supervised description task is feasible for collecting sociophonetic decision-based perceptual data. A similar method with the same parameters (descriptors) as this study also showed potential capacity for correctly classifying participants into dialectal groups based on their descriptive results (Gibbon and Liu, 2018).

The task followed a supervised description procedure in which participants were required to describe a heard sound based on provided descriptions. However, the results cannot be unilaterally defined as perceptual. During the task phases, the participants had to make an acoustic decision on an acoustic feature of the sound, which was suspected to have triggered the motor procedures for production. The results from the present experiment showed high awareness of the participants in respect of the acoustic decision they were making. If a further step is taken to ask the listeners to imitate the sound, these acoustic decisions may affect the production automatically. Researchers found an automatic relationship between perception and production (Goldinger, 1998; Shockley et al., 2004; Pickering and Garrod, 2013; Walker and Campbell-Kibler, 2015). Therefore, the description task here is more than merely perception and less than real production. It is quite faithful to the acoustic awareness of the listeners.

## Conclusion

This study investigates tonal variant descriptions of the official dialect in China (Putonghua) as a factor in the coevolution of dialects. Three sociophonetic factors, *target tone familiarity, tonal variant familiarity*, and *tonal inventory size*, are included in order to raise interesting theoretical questions concerning the role of familiarity and dialect experience in sound change. Standard Putonghua tones are manipulated in terms of height and shape as stimuli related to tonal variants. Speakers from three Chinese dialect groups, BM, SW, and GC, are invited to rate the applicability of the designed description of pitch contour and height to the stimuli. The three dialects have different tonal inventory sizes, and their native speakers have different levels of familiarity with Putonghua tones or with Putonghua tonal variants related to the category-to-category assimilation between native dialect tone and the stimuli. Difference in the above three sociophonetic factors is predicts differently in listeners' performance.

The GC speakers outperform the BM and SW speakers in describing Putonghua tone height variants, while the BM speakers showed high proficiency and marginally better performance than the SW dialect speakers. Both findings confirm the relevance of *tonal variant familiarity*, which refers to category-to-category assimilation between the native dialect tone and the stimuli in predicting participants' descriptive decisions. Familiarity with the phonetic feature of the tone patterns of the target language was also combined with dialect experience to explain the successful descriptions of tone shape variants. Clearly, care must be exercised in drawing further conclusions, since the study only took Chinese east coast city dialects into consideration, and further studies must consider the western dialects from north to south, as well as differences between city and rural dialects.

While the pattern observed in this study is a small part of how sound change is judged in the acoustic decision system, it cannot predict which judgments would trigger sound changes eventually. Thus, in future studies, mechanisms of how sociophonetic-related acoustic decisions trigger sound changes in production, especially in an interactive context, are in urgent need of investigation. The findings and methodology reported in this study will be crucial in future attempts to explore the relevance between sociophonetic factors and listeners' acoustic decisions on cross-dialect tonal variations.

## Data Availability Statement

The raw data supporting the conclusions of this article will be made available by the authors, without undue reservation.

## Ethics Statement

Ethical review and approval was not required for the study on human participants in accordance with the local legislation and institutional requirements. Written informed consent for participation was not required for this study in accordance with the national legislation and the institutional requirements.

## Author Contributions

HL and DG: conceptualization, validation, methodology, writing–review and editing, visualization, and supervision. DG: software and formal analysis. HL: investigation, resources, data curation, writing-original draft, project administration, and funding acquisition. All authors contributed to the article and approved the submitted version.

## Funding

This work was supported by Humanities and Social Sciences Youth Foundation, Ministry of Education of the People's Republic of China (Grant No: 21YJC740035).

## Conflict of Interest

The authors declare that the research was conducted in the absence of any commercial or financial relationships that could be construed as a potential conflict of interest.

## Publisher's Note

All claims expressed in this article are solely those of the authors and do not necessarily represent those of their affiliated organizations, or those of the publisher, the editors and the reviewers. Any product that may be evaluated in this article, or claim that may be made by its manufacturer, is not guaranteed or endorsed by the publisher.

## Figures and Tables

**Figure A1:**
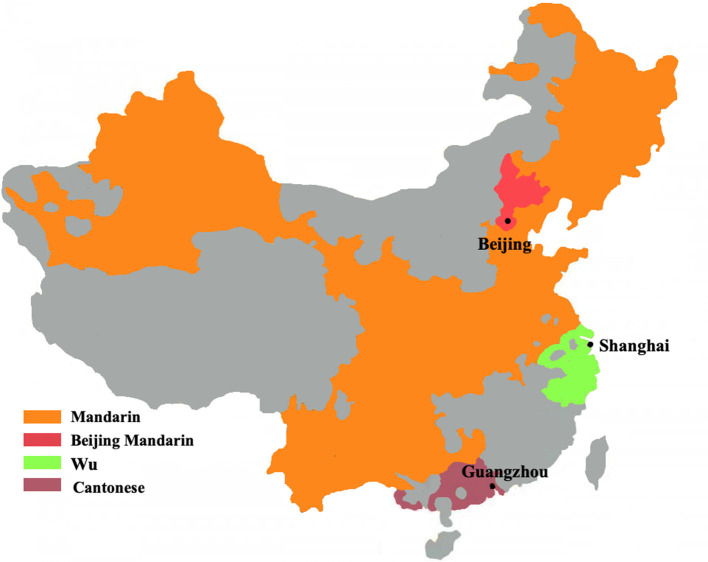
Locations of Beijing Mandarin, Shanghai Wu, and Guangzhou Cantonese.

## Footnotes

^1^Studies on natural language processing have given quite a lot of evidence to low flat variants for T2 and T4 (Zhu, 1999; Rong et al., 2015). The low flat variants for T2 and T4 are more likely to occur in a sociophonetic context, such as uncared utterances or rapid oral responses, rather than in a standard linguistic context.

^2^The Shang tone of Putonghua is a low flat tone by nature (Shi and Ran, 2011). Previous studies have shown that listeners follow a categorical perception when distinguishing *Shang* and *Yangpin*, as well as *Shang* and *Qu*. Yu (2019) asked listeners to identify *Shang*/*Yangpin* on a continuum from a low flat tone to a rising tone, and to identify *Shang*/Qu on a continuum from a low flat tone to a falling tone. The listeners showed a strong pattern of categorical perception in both tasks. The same conclusion has also been drawn by Wu (2011), Wang and Qin (2015), and Qi et al. (2016). Therefore, here, the stimuli of set 3 for T2 and T4, as a low flat tone, should show categorical differences with the original tones.
